# Supplementary material for: The Unfolded Protein Response Is Associated with Cancer Proliferation and Worse Survival in Hepatocellular Carcinoma
Source: Cancers (Basel). 2021 Sep 3;13(17):4443. doi: 10.3390/cancers13174443 (PMC8430652; doi:10.3390/cancers13174443)
Supplement: Supplementary file 1 [file cancers-13-04443-s001.zip › cancers-1251776-supplementary.pdf]

cancers

*Supplementary Files*

# The Unfolded Protein Response Is Associated with Cancer Proliferation and Worse Survival in Hepatocellular Carcinoma

**Ankit Patel, Masanori Oshi, Li Yan, Ryusei Matsuyama, Itaru Endo and Kazuaki Takabe**

**Table S1.** Member genes of the Unfolded protein response score.

| Gene Name | Gene Description |
| --- | --- |
| *ALDH18A1* | aldehyde dehydrogenase 18 family member A1 |
| *ARFGAP1* | ADP ribosylation factor GTPase activating protein 1 |
| *ASNS* | asparagine synthetase |
| *ATF3* | activating transcription factor 3 |
| *ATF4* | activating transcription factor 4 |
| *ATF6* | activating transcription factor 6 |
| *ATP6V0D1* | ATPase H+ transporting V0 subunit d1 |
| *BAG3* | BAG cochaperone 3 |
| *BANF1* | BAF nuclear assembly factor 1 |
| *CALR* | calreticulin |
| *CCL2* | C-C motif chemokine ligand 2 |
| *CEBPB* | CCAAT enhancer binding protein beta |
| *CEBPG* | CCAAT enhancer binding protein gamma |
| *CHAC1* | ChaC glutathione specific gamma-glutamylcyclotransferase 1 |
| *CKS1B* | CDC28 protein kinase regulatory subunit 1B |
| *CNOT2* | CCR4-NOT transcription complex subunit 2 |
| *CNOT4* | CCR4-NOT transcription complex subunit 4 |
| *CNOT6* | CCR4-NOT transcription complex subunit 6 |
| *CXXC1* | CXXC finger protein 1 |
| *DCP1A* | decapping mRNA 1A |
| *DCP2* | decapping mRNA 2 |
| *DCTN1* | dynactin subunit 1 |
| *DDIT4* | DNA damage inducible transcript 4 |
| *DDX10* | DEAD-box helicase 10 |
| *DKC1* | dyskerin pseudouridine synthase 1 |
| *DNAJA4* | DnaJ heat shock protein family (Hsp40) member A4 |
| *DNAJB9* | DnaJ heat shock protein family (Hsp40) member B9 |
| *DNAJC3* | DnaJ heat shock protein family (Hsp40) member C3 |
| *EDC4* | enhancer of mRNA decapping 4 |
| *EDEM1* | ER degradation enhancing alpha-mannosidase like protein 1 |
| *EEF2* | eukaryotic translation elongation factor 2 |
| *EIF2AK3* | eukaryotic translation initiation factor 2 alpha kinase 3 |
| *EIF2S1* | eukaryotic translation initiation factor 2 subunit alpha |
| *EIF4A1* | eukaryotic translation initiation factor 4A1 |
| *EIF4A2* | eukaryotic translation initiation factor 4A2 |
| *EIF4A3* | eukaryotic translation initiation factor 4A3 |
| *EIF4E* | eukaryotic translation initiation factor 4E |
| *EIF4EBP1* | eukaryotic translation initiation factor 4E binding protein 1 |
| *EIF4G1* | eukaryotic translation initiation factor 4 gamma 1 |
| *ERN1* | endoplasmic reticulum to nucleus signaling 1 |
| *ERO1L* | endoplasmic reticulum oxidoreductase 1 alpha |
| *EXOC2* | exocyst complex component 2 |
| *EXOSC1* | exosome component 1 |
| *EXOSC10* | exosome component 10 |
| *EXOSC2* | exosome component 2 |
| *EXOSC4* | exosome component 4 |
| *EXOSC5* | exosome component 5 |

| | |
|---|---|
| *EXOSC9* | exosome component 9 |
| *FKBP14* | FKBP prolyl isomerase 14 |
| *FUS* | FUS RNA binding protein |
| *GEMIN4* | gem nuclear organelle associated protein 4 |
| *GOSR2* | golgi SNAP receptor complex member 2 |
| *H2AFX* | H2A.X variant histone |
| *HERPUD1* | homocysteine inducible ER protein with ubiquitin like domain 1 |
| *HSP90B1* | heat shock protein 90 beta family member 1 |
| *HSPA5* | heat shock protein family A (Hsp70) member 5 |
| *HSPA9* | heat shock protein family A (Hsp70) member 9 |
| *HYOU1* | hypoxia up-regulated 1 |
| *IARS* | isoleucyl-tRNA synthetase 1 |
| *IFIT1* | interferon induced protein with tetratride repeats 1 |
| *IGFBP1* | insulin like growth factor binding protein 1 |
| *IMP3* | IMP U3 small nucleolar ribonucleoprotein 3 |
| *KDELR3* | KDEL endoplasmic reticulum protein retention receptor 3 |
| *KHSRP* | KH-type splicing regulatory protein |
| *KIF5B* | kinesin family member 5B |
| *LSM1* | LSM1 homolog, mRNA degradation associated |
| *LSM4* | LSM4 homolog, U6 small nuclear RNA and mRNA degradation associated |
| *MTHFD2* | methylenetetrahydrofolate dehydrogenase (NADP+ dependent) 2 |
| *NFYA* | nuclear transcription factor Y subunit alpha |
| *NFYB* | nuclear transcription factor Y subunit beta |
| *NHP2* | NHP2 ribonucleoprotein |
| *NOLC1* | nucleolar and coiled-body phosphoprotein 1 |
| *NOP14* | NOP14 nucleolar protein |
| *NOP56* | NOP56 ribonucleoprotein |
| *NPM1* | nucleophosmin 1 |
| *OBFC2A* | nucleic acid binding protein 1 |
| *PAIP1* | poly(A) binding protein interacting protein 1 |
| *PARN* | poly(A)-specific ribonuclease |
| *PDIA5* | protein disulfide isomerase family A member 5 |
| *PDIA6* | protein disulfide isomerase family A member 6 |
| *POP4* | POP4 homolog, ribonuclease P/MRP subunit |
| *PREB* | prolactin regulatory element binding |
| *PSAT1* | phosphoserine aminotransferase 1 |
| *RPS14* | ribosomal protein S14 |
| *RRP9* | ribosomal RNA processing 9, U3 small nucleolar RNA binding protein |
| *SDAD1* | SDA1 domain containing 1 |
| *SEC11A* | SEC11 homolog A, signal peptidase complex subunit |
| *SEC31A* | SEC31 homolog A, COPII coat complex component |
| *SERP1* | stress associated endoplasmic reticulum protein 1 |
| *SHC1* | SHC adaptor protein 1 |
| *SKIV2L2* | Mtr4 exosome RNA helicase |
| *SLC1A4* | solute carrier family 1 member 4 |
| *SLC30A5* | solute carrier family 30 member 5 |
| *SLC7A5* | solute carrier family 7 member 5 |
| *SPCS1* | signal peptidase complex subunit 1 |
| *SPCS3* | signal peptidase complex subunit 3 |
| *SRPR* | SRP receptor subunit alpha |
| *SRPRB* | SRP receptor subunit beta |
| *SSR1* | signal sequence receptor subunit 1 |
| *STC2* | stanniocalcin 2 |
| *TARS* | threonyl-tRNA synthetase 1 |
| *TATDN2* | TatD DNase domain containing 2 |
| *TSPYL2* | TSPY like 2 |
| *TTC37* | tetratricopeptide repeat domain 37 |
| *TUBB2A* | tubulin beta 2A class IIa |
| *VEGFA* | vascular endothelial growth factor A |
| *WFS1* | wolframin ER transmembrane glycoprotein |

| | |
|---|---|
| *WIPI1* | WD repeat domain, phosphoinositide interacting 1 |
| *XBP1* | X-box binding protein 1 |
| *XPOT* | exportin for tRNA |
| *YIF1A* | Yip1 interacting factor homolog A, membrane trafficking protein |
| *YWHAZ* | tyrosine 3-monooxygenase/tryptophan 5-monooxygenase activation protein zeta |
| *ZBTB17* | zinc finger and BTB domain containing 17 |

**Table S2.** Univariate and multivariate analysis with overall survival in the TCGA cohort.

| | **HR** | **95%CI** | | ***p*** | | **HR** | **95%CI** | | ***p*** | |
|---|---|---|---|---|---|---|---|---|---|---|
| >60 vs. 60 ≥ | 1.26 | 0.89 | 1.79 | 0.189 | | | | | | |
| WHITE vs. Others | 1.24 | 0.87 | 1.76 | 0.238 | | | | | | |
| III/IV vs. I/II | 2.40 | 1.65 | 3.48 | <0.001 | * | 2.19 | 1.50 | 3.20 | <0.001 | * |
| G3/G4 vs. G1/G2 | 1.11 | 0.78 | 1.58 | 0.565 | | | | | | |
| High vs. Low | 1.98 | 1.39 | 2.82 | <0.001 | * | 1.59 | 1.09 | 2.32 | 0.016 | * |

**Table S3.** Cox regression analysis of cell proliferation-related gene sets.

| **DFS** | **HR** | **95%CI** | | ***p*** |
|---|---|---|---|---|
| *UPR* | 3.45 | 1.81 | 6.60 | <0.001 |
| *E2F* | 2.53 | 1.69 | 3.78 | <0.001 |
| *G2M* | 2.58 | 1.68 | 3.95 | <0.001 |
| *MYC v1* | 3.08 | 1.90 | 5.00 | <0.001 |
| *MYC v2* | 2.89 | 1.80 | 4.65 | <0.001 |
| *MITOTIC* | 2.21 | 1.33 | 3.69 | 0.002 |
| *MKI67* | 1.82 | 1.30 | 2.54 | <0.001 |
| **DSS** | **HR** | **95%CI** | | ***p*** |
| *UPR* | 6.21 | 2.65 | 14.55 | <0.001 |
| *E2F* | 3.65 | 2.07 | 6.44 | <0.001 |
| *G2M* | 3.86 | 2.12 | 7.03 | <0.001 |
| *MYC v1* | 4.77 | 2.46 | 9.25 | <0.001 |
| *MYC v2* | 3.32 | 1.76 | 6.27 | <0.001 |
| *MITOTIC* | 3.27 | 1.63 | 6.55 | <0.001 |
| *MKI67* | 2.43 | 1.53 | 3.86 | <0.001 |
| **OS** | **HR** | **95%CI** | | ***p*** |
| *UPR* | 5.67 | 2.91 | 11.05 | <0.001 |
| *E2F* | 2.73 | 1.78 | 4.20 | <0.001 |
| *G2M* | 2.85 | 1.81 | 4.51 | <0.001 |
| *MYC v1* | 3.61 | 2.17 | 6.00 | <0.001 |
| *MYC v2* | 2.87 | 1.75 | 4.72 | <0.001 |
| *MITOTIC* | 2.44 | 1.43 | 4.16 | 0.001 |
| *MKI67* | 1.27 | 1.15 | 1.42 | <0.001 |

**Table S4.** List of Software used in analysis.

| **Software** | **Version** | **Reference** |
|---|---|---|
| R | 4.1.0 | - |
| GSEA | 4.1.0 | Subramanian A, et. al. Gene set enrichment analysis: a knowledge-based approach for interpreting genome-wide expression profiles. Proc Natl Acad Sci U S A. 2005 Oct 25;102(43):15545-50. doi: 10.1073/pnas.0506580102. Epub 2005 Sep 30. |
| GSVA | 1.38.2 | Hänzelmann S, et. al. GSVA: gene set variation analysis for microarray and RNA-seq data. BMC Bioinformatics. 2013 Jan 16;14:7. doi: 10.1186/1471-2105-14-7. |
| Microsoft Excel | 16.0 | Microsoft Windows 10 |

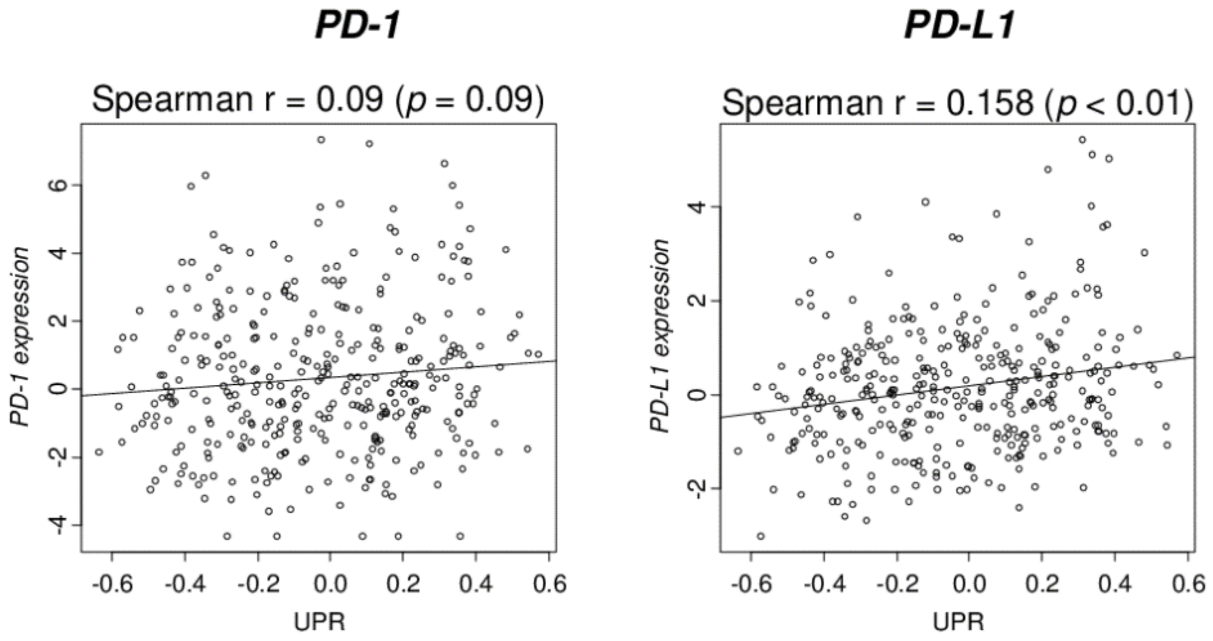


**Figure S1.** Co-relation curve of PD-1, PDL-1 expressions and UPR score.
